# Improving image classification of gastrointestinal endoscopy using curriculum self-supervised learning

**DOI:** 10.1038/s41598-024-53955-8

**Published:** 2024-03-13

**Authors:** Han Guo, Sai Ashish Somayajula, Ramtin Hosseini, Pengtao Xie

**Affiliations:** Department of Electrical and Computer Engineering, University of California, San Diego, San Diego, 92093 USA

**Keywords:** Ulcers, Endoscopy, Diagnosis

## Abstract

Endoscopy, a widely used medical procedure for examining the gastrointestinal (GI) tract to detect potential disorders, poses challenges in manual diagnosis due to non-specific symptoms and difficulties in accessing affected areas. While supervised machine learning models have proven effective in assisting clinical diagnosis of GI disorders, the scarcity of image-label pairs created by medical experts limits their availability. To address these limitations, we propose a curriculum self-supervised learning framework inspired by human curriculum learning. Our approach leverages the HyperKvasir dataset, which comprises 100k unlabeled GI images for pre-training and 10k labeled GI images for fine-tuning. By adopting our proposed method, we achieved an impressive top-1 accuracy of 88.92% and an F1 score of 73.39%. This represents a 2.1% increase over vanilla SimSiam for the top-1 accuracy and a 1.9% increase for the F1 score. The combination of self-supervised learning and a curriculum-based approach demonstrates the efficacy of our framework in advancing the diagnosis of GI disorders. Our study highlights the potential of curriculum self-supervised learning in utilizing unlabeled GI tract images to improve the diagnosis of GI disorders, paving the way for more accurate and efficient diagnosis in GI endoscopy.

## Introduction

The gastrointestinal (GI) tract is susceptible to a wide range of disorders and conditions. These conditions can cause a variety of symptoms, including abdominal pain, bloating, changes in bowel habits, difficulty swallowing, and gastrointestinal bleeding^1–3^. For instance, according to the National Institute of Diabetes and Digestive and Kidney Disease (NIDDK), more than 60 million people are affected by medical conditions related to GI tract. Diseases associated with the GI tract were responsible for a crude rate of 37.2 deaths per 100,000 population in the region of Americas in 2019^4^.

Currently, endoscopy is the standard procedure for examining the GI tract. Endoscopic examinations allow for direct visualization of internal organs, tissues, and cavities, facilitating the accurate detection of abnormalities such as tumors, ulcers, inflammation, and other pathologies^5^. This aids in early detection and timely intervention, leading to more effective treatment strategies and improved patient prognosis. Moreover, endoscopy allows for ongoing monitoring and surveillance of chronic conditions, such as inflammatory bowel disease, Barrett’s esophagus, and chronic stomach disease^6^. Repeated endoscopic examinations enable physicians to assess treatment efficacy, disease progression, and response to therapy, guiding subsequent treatment decisions. Endoscopy is commonly used in gastroenterology, pulmonology, gynecology, urology, and other medical specialties^7–11^. It offers numerous advantages over traditional surgical methods, including shorter recovery times, reduced risks of complications, and minimal scarring^1,12^.

However, diagnosing GI diseases using endoscopy is challenging due to limited view and overlapping conditions that humans are prone to overlook^13^. On the other hand, the advancement of deep learning has proven successful in multiple computer vision (CV) tasks, including image classification and semantic segmentation, with accuracy comparable or even superior to human experts^14^. Given its empirical success in CV tasks, deep learning has been adopted in the healthcare domain to assist physicians in both research and clinical diagnosis. Neural network (NN) based models have already been widely utilized in numerous medical imaging problems, including brain tumor classification^15^, lung segmentation^16^, and endoscopic anomaly detection^17^. In particular, machine learning models can be trained to analyze images or videos taken from an endoscopy and identify subtle abnormalities or patterns that may be difficult for human observers to detect. Traditionally, this is done in a supervised setting where NN models are trained on endoscopic datasets with image-text pairs. However, this approach presents challenges due to the extensive human effort required by experienced physicians to label the pathological conditions and anatomical landmarks observed during endoscopy. In contrast, there is an abundance of unlabeled endoscopic data available. For example, HyperKvasir dataset contains 10 times more unlabeled GI endoscopy images than labeled ones^18^. Therefore, harnessing both labeled and unlabeled data becomes a more preferable strategy compared to training solely on the labeled data. By incorporating unlabeled data into the training process, we can potentially enhance the generalization and robustness of the models, leading to improved diagnostic capabilities in endoscopy.

Alternatively, self-supervised methods leverage unlabeled data to learn useful representations without explicit human annotations. To accomplish this, pretext tasks are used in self-supervised learning as surrogate tasks that indirectly capture meaningful patterns in the data^19^. By solving these pretext tasks, models can learn to extract informative features and structures^20,21^. Data augmentation plays a critical role in this process because it increases the diversity and variability of the training data, enabling the model to learn robust representations that generalize well to unseen examples^22^. It achieves this by introducing various transformations to the input data, providing different perspectives and helping the model learn invariant representations^23,24^. Insufficient augmentation may hinder model convergence, while overly strong augmentation can introduce unwanted noise during training^22^. To fully leverage the potential of self-supervised learning on endoscopic datasets, we propose Curriculum Mixup (C-Mixup), a framework that incorporates curriculum learning and Mixup as data augmentation methods using contrastive learning. C-Mixup modifies the data augmentation pipeline with a curriculum scheduler and image mixture process, mitigating the negative impact of additive noise from strong augmentations. Our models are trained on a modified HyperKvasir^18^ dataset, comprising 99,148 unlabeled GI endoscopy images and 10,490 labeled images categorized into 16 classes based on anatomical landmarks, pathological findings, and normal findings. The empirical results demonstrate the effectiveness of our method by achieving 88.92% top-1 accuracy in the endoscopic image classification task, a 2.1% increase from the vanilla SimSiam baseline. The contribution of our works are the following

- We evaluate SOTA self-supervised learning methods on endoscopic dataset, and show that vanilla self-supervised methods do not yield desirable performance.
- Our work explores the theoretical and empirical setup that jointly represents C-Mixup using one of the SOTA self-supervised method, SimSiam.
- To the best of our knowledge, our work is the first to propose using curriculum learning and Mixup as the data augmentation method to further boost the performance in the self-supervised learning paradigm on endoscopic dataset.

## Related work

### Endoscopic image classification

Over the past few years, the landscape of endoscopic image classification has been transformed through the emergence of larger, more refined datasets and deep learning models. Initially, the endoscopic image classification was conducted using pre-defined rules. For example, Wang et al.^25^ proposed a software system that detects polyps via edge-cross-section visual features and a rule-based classifier that enables the tracking of the same polyp edge in a sequence of images. The evolution from rule-based systems to more complex deep learning models marked a pivotal shift in the field. Gamage et al.^26^ implemented an ensemble of DenseNet-201 with an artificial neural network to classify various digestive tract diseases, achieving a significant accuracy boost. Similarly, Takiyama et al.^27^ utilized a GoogLeNet-based approach to automatically classify anatomical structures in thousands of esophagogastroduodenoscopy images, demonstrating high accuracy in identifying key gastrointestinal regions. The development continued with Shichijo et al.^28^ and Byrne et al.^29^, who each trained Convolutional Neural Network (CNN) models for specific diagnostic purposes, with the latter focusing on real-time assessment of colorectal polyps using narrow-band imaging video frames. Zhang et al.^30^ introduced an innovative approach by employing transfer learning with a CNN trained on non-medical images to facilitate knowledge transfer from non-medical domains to endoscopy, significantly reducing the dependency on extensive labeled medical data. Their method allows for efficient knowledge transfer from non-medical domains to the medical field, reducing the need for huge sizes of labeled medical data. More recent advancements have focused on refining these deep-learning models to address specific challenges within the field. Song et al.^31^ developed a computer-aided diagnostic system with a 50-layer convolutional neural network that performs comparably to human experts in colorectal polyp histology prediction. Yue et al.^32^ introduced novel loss formulation strategies to tackle class imbalance and hard sampling problems. To avoid paying excessive attention to the junction of the digestive tract, Wang et al.^33^ combined CNN with a capsule network, incorporating lesion-aware feature extraction to improve focus on relevant areas. Furthering the innovation, Mohapatra et al.^34^ proposed using empirical wavelet transform to extract frequency components from endoscopic data before applying a CNN model for training and testing. Luo et al.^35^ proposed UC-DenseNet, which combines CNN and RNN along with an improved attention mechanism to emphasize feature information through cross-channel communication. All these works require image-label pairs and do not leverage the large unlabeled endoscopic dataset that is available. We propose to incorporate the self-supervised learning strategy to tackle the endoscopic image classification task.

### Self-supervised learning and its application on gastrointestinal endoscopy

Self-supervised learning (SSL) has emerged as a transformative approach in computer vision, demonstrating significant empirical success across various tasks, including image classification^22,23,36,37^, semantic segmentation^36,38,39^, and object detection^40,41^. This method has been particularly beneficial in medical imaging, where labeled data can be scarce and expensive to obtain^42,43^.

SSL has been leveraged in the critical area of endoscopic depth estimation, a task distinct from endoscopic image classification, focusing on spatial depth perception rather than categorizing visual content. For instance, Shao et al.^44^ introduced a self-supervised learning framework specifically for depth and ego-motion estimation in endoscopic videos, leveraging a novel concept called ’appearance flow’ to account for brightness variations in these images. Similarly, Liu et al.^45^ also explored self-supervised learning, focusing on depth and pose estimation in gastrointestinal endoscopy. Their model, which includes networks for both depth and pose estimation, leverages self-supervised training. This is achieved through a multi-scale structural similarity combined with L1 norm (MS-SSIM+L1) loss, calculated between the target frame and the reconstructed image, showcasing the applicability of SSL in complex medical imaging tasks. Another work employs a self-supervised dual-branch Siamese network, leveraging sparse self-supervisory signals from Structure from Motion (SfM) for dense depth prediction. Sparse Flow Loss and Depth Consistency Loss guide the network to produce accurate, smooth depth maps by utilizing sparse reconstructions and geometric constraints^46^.

SSL also shows potentials in endoscopic image matching and video analysis. While our work focuses on categorizing images into predefined classes, image matching and video analysis involves tasks like extracting distinct visual features, aligning similar images, and temporal data analysis. Farhat et al.^47^ introduced a SSL based approach on raw video frames to train a CNN-based model for keypoint matching in endoscopic images. Central to its training is a triplet loss architecture that utilizes raw video frames instead of labeled data. Ross et al.^48^ introduced the Pre-training with Auxiliary Task (PAT) method that falls under the umbrella of SSL. This method utilizes large amounts of unlabeled endoscopic video data to boost CNN performance in medical imaging tasks like instrument segmentation. Pascual et al.^49^ devised a two-stage process utilizing SSL to extract meaningful information from unlabeled endoscopic video data. In the first stage, the model uses the temporal sequence of images in the videos to generate embeddings, employing per-frame pseudo-labels and a triplet loss contrastive learning mechanism. In the second stage, these embeddings are finetuned with limited labeled data for specific medical tasks, using a combination of softmax cross-entropy loss and Triplet Loss in a ResNet-50 based architecture.

Although SSL has demonstrated effectiveness in general computer vision tasks and certain aspects of gastrointestinal endoscopy, its specific application in the nuanced field of endoscopic classification, especially with the latest contrastive learning methods, is still an area ripe for exploration. The only study we are aware of in this area is by Huang et al.^50^, which focused on using SimCLR^22^, an SSL method. This method maximizes agreement between differently augmented views of the same data instance in a latent space and requires an extremely large batch size to avoid collapsing. It was used to classify polyps in endoscopic images, specifically colorectal polyps in Blue Laser Imaging (BLI) images. However, their method is less suitable for classifying more diffuse conditions like esophagitis, which typically appears across various areas, and is challenging to apply their method which requires localization.

While in traditional contrastive learning, both positive and negative samples are required, a recent framework, SimSiam^36^, completely abandoned the negative samples during the visual representation process by introducing a siamese network with pairwise augmented views. Although some works show the potential importance of negative samples^51–54^, they all require the negative sample to be “true negative sample”, i.e. the negative sample must be in a different class than the positive sample. For example, Pacal et al.^51^ required polyp-free images as negative samples used during training to counteract the effect of false alarms by using images that do not contain polyps. Similarly, Wang et al.^54^ required images containing sessile polyps to be positive while images containing pedunculated polyps to be negative. All these works that leveraged the negative samples require the true class labels, which cannot be obtained during the pretraining using unlabeled data. In contrastive learning, the negative sample is defined to be any instance that is different from the current anchor image, even though the anchor image and the negative sample belong to the same class. Furthermore, Awasthi et al.^55^ showed an ultimate collision-coverage trade-off of having more negative examples and hurting the downstream performance. In addition, some works theoretically showed the advantage of SimSiam which does not use negative sampling while maintaining comparable or better performance across various computer vision tasks^56,57^. Given these observations, we employ the SimSiam approach in our method.

## Methods

In this section, we will introduce C-Mixup, a generic data augmentation strategy inspired by curriculum learning and image mixture process on contrastive learning framework. Specifically, we utilize Mixup and SimSiam framework to accomplish our design that is effective and robust to classify GI conditions based on the data from the endoscopy.

### Mixup

Mixup^58^ is a generic vicinal distribution that produce virtual feature-target pairs from,

1 $$\begin{aligned} \mu (\tilde{x}, \tilde{y}|x_i,y_i)=\frac{1}{n}\displaystyle \sum _j^n\mathbb {E}_{\lambda }[\delta (\tilde{x} = \lambda x_i + (1-\lambda )x_j, \tilde{y} = \lambda y_i + (1-\lambda )y_j)] \end{aligned},$$

where $\delta (x=x_i, y=y_j)$ is a Dirac mess centered at $(x_i,y_j)$. Thus, given input vectors $x_i, x_j$ and target vectors $y_i,y_j$, the corresponding virtual feature-target pair is defined as,

$$\tilde{x} = \lambda x_i + (1-\lambda )x_j$$

$$\tilde{y} = \lambda y_i + (1-\lambda )y_j$$

where $\lambda \sim \text {Beta}(\alpha , \alpha )$ for $\alpha \in (0, \infty )$. This image mixture process can be seen as a special kind of data augmentation technique. By producing linearly in-between virtual samples, Mixup reduces oscillations and provides smoother predictions on data outside training samples^58^.

### SimSiam

Our experiments are conducted using one of the contrastive learning methods, SimSiam^36^. Contrastive learning, in general, is a self-supervised pre-training paradigm where visual representation is learned without ground-truth labels. In specific, SimSiam takes two randomly augmented views $x_1, x_2$ from the same input image *x*. Both augmented views are then passed through a shared-weight encoder, which is a deep neural network e.g. ResNet50, and a projection MLP layer. Augmented view $x_1$ will pass an additional prediction MLP head which transforms to the same dimension as $x_2$. Denote the encoder plus projection MLP as *f* and prediction MLP as *h* and denote two output vectors as $p_1\overset{\Delta }{=} h(f(x_1))$ and $z_2\overset{\Delta }{=}\ f(x_2)$. The training objective is to minimize the negative cosine similarity between $p_1, z_2$

2 $$\begin{aligned} \mathfrak {L} = -\frac{1}{2}\frac{p_1}{\Vert p_1\Vert _2}\cdot \text {StopGrad}(\frac{z_2}{\Vert z_2\Vert _2}) -\frac{1}{2}\frac{p_2}{\Vert p_2\Vert _2}\cdot \text {StopGrad}(\frac{z_1}{\Vert z_1\Vert _2}) \end{aligned},$$

where $\Vert \cdot \Vert _2$ is *l*2 norm and $\text {StopGrad}(\cdot )$ is the stop-gradient operation. The negative cosine similarity is calculated twice by which each view is applied with the stop-gradient operation once.

### Curriculum-Mixup as data augmentation in SimSiam

In contrastive learning, the formation of contrastive pairs is critical for models to learn good visual representations since the goal is to encourage augmentations (views) of the same source image to have more similar representations and different images to have dissimilar representations. InfoMin^59^ demonstrates that a good view in a positive pair should contain intact task-relevant information while reducing the mutual information (MI) between two views. Formally, let $v_1, v_2$ be two views, *y* be prediction, and $I(v_1;v_2)$ be information shared between $v_1$ and $v_2$. InfoMin defines the optimal positive pair when $I(v_1;y) = I(v_2;y)$, meaning $v_1, v_2$ only share task related information. In other words, two views in a positive pair should both include the target object while keeping the background as diverse/different as possible. Following this concept, SimSiam requires strong data augmentation to minimize the MI between two augmented views. Yet, excessive data augmentation also disturbs the training process since strong augmentation typically introduces noises, resulting in learning suboptimal visual features.

The optimization objective is to minimize the negative cosine similarity between two augmented views. However, each augmented view goes through the data augmentation separately and the stochasticity of applying certain transformations complicate the cosine similarity between two augmented views. To have controllable cosine similarity during the pre-training process, we seek a solution from the Mixup operation. Yet the tradeoff of high MI and large noise exists. To tackle this problem, we incorporate the design of curriculum learning.

The idea of curriculum learning is inspired by how humans learn, starting with simpler concepts before moving on to more complex ones. By gradually increasing the complexity of the examples presented to the model during training, the model can better learn and generalize from the data. One advantage of curriculum learning is that it can help models avoid getting stuck in local optima or overfitting to the training data. By starting with simpler examples, the model can build a strong foundation before moving on to more complex examples, which can help it avoid getting stuck in local optima. Empirically, it has been shown that curriculum learning helps the model to train better in a noisy setting^60^. Based on this insight, we propose Curriculum-Mixup, a progressive training framework for self-supervised learning. Curriculum-Mixup (C-Mixup) aims to enhance the data augmentation in the contrastive learning pipeline by generating more meaningful augmented views with a hardness-aware augmentation method. In our framework, we define a curriculum order on the strength of the data augmentation. Our eventual goal is to let the contrastive model learn a better representation by utilizing the hardness-aware augmentation method and the curriculum learning strategy.

Different from the classical curriculum learning setting where the training samples are ordered and trained in a easy to difficult fashion defined by the training loss^61^, our method defines difficulty in the data pre-processing stage. In our C-Mixup setting, the difficulty is defined by the magnitude of the Mixup. Since the optimization goal in Eq. (2) is to minimize the negative cosine similarity between the prediction given the input augmented view $x_i$ and the ground-truth augmented view $x_j$, the similar $x_i$ is to $x_j$, the easier the prediction task is. Therefore we define a easier task is that $x_i$ and $x_j$ are similar to each other and a harder task is that $x_i$ and $x_j$ are dissimilar to each other. To control the similarity between $x_i$ and $x_j$, we define $\tilde{x}_j$ to be a mixture by $x_i$ and $x_j$ and $(x_i, \tilde{x}_j)$ to be the new augmented view pair. In nutshell, the trivial case will be $\tilde{x}_j=x_i$ and the augmented view pair will be $(x_i, x_i)$. Conversely, the hardest case will be $\tilde{x}_j = x_j$ and the augmented view pair will be $(x_i, x_j)$. The hardness of the similarity is controlled by the Mixup $\lambda$.

Inspired by Curriculum Dropout^62^, we propose a curriculum function $\lambda (t)$ that controls the Mixup parameter $\lambda$ with input of current training iteration *t*. Let $\lambda _{max}$ and $\lambda _{min}$ be upper and lower limit of $\lambda$, any function that $t\mapsto \lambda (t)$ such that $\lambda (0)=\lambda _{max}$ and $\displaystyle \lim _{t\rightarrow \infty } \lambda (t) =\lambda _{min}$ is said to be a curriculum function bounded by $\lambda _{min}$ and $\lambda _{max}$. Starting from $\lambda (0)=\lambda _{max}$ where $\tilde{x}_j$ consists the maximal of $x_i$ within the boundary, $\tilde{x}_j$ gradually reduces its composition of $x_i$ in a way that $\lambda (t)\ge \lambda _{min}$. At the end of the training, $\lambda _(t)\rightarrow \lambda _{min}$ is equivalent to the original formulation of fixed Mixup training. Although the choices of $\lambda (t)$ are not limited as long as $\lambda (t)$ is monotonically decreasing, in our method, we adopt step function.

3 $$\begin{aligned} \lambda _{curriculum}(t)=\lambda _{max}-(\frac{(\lambda _{max}-\lambda _{min})}{step\_size}\cdot \lfloor \frac{i\cdot step\_size}{t}\rfloor + \lambda _{min}) \end{aligned},$$

In Eq. (3), we define $\lambda _{max}$, $\lambda _{min}$ as the upper and lower boundary of the Mixup lambda value. We also define $step\_size$ to constrain the update frequency of the Mixup lambda. Intuitively, we update the Mixup lambda value in an equal interval determined by $step\_size$ and total epoch *t*. A detailed discussion is presented in the ablation section.

Formally, given two randomly augmented views $x_i, x_j$ from *x*, we define the positive view of $x_i$ to be

4 $$\begin{aligned} \tilde{x}_j = \lambda x_i+(1-\lambda )x_j \end{aligned},$$

**Figure 1:**
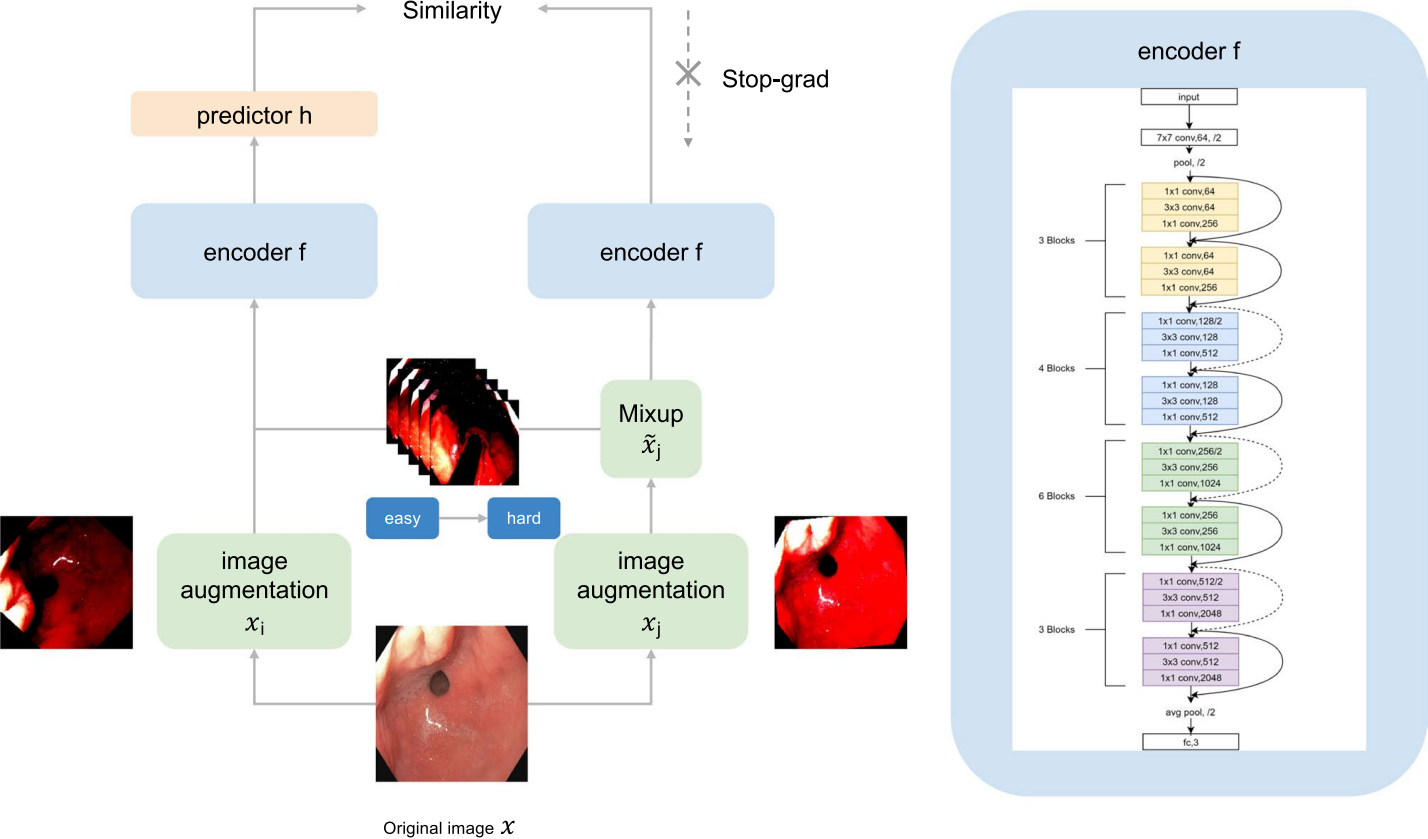
Overview of the proposed C-Mixup method. The C-Mixup method begins by generating two augmented views, $x_i$ and $x_j$, from the input image. Next, we apply the Mixup operation to $x_j$ using $x_i$, creating a mixed view $\tilde{x_j}$. Subsequently, view $x_i$ goes through a backbone encoder and predictor, which generate predictions for the encoded $\tilde{x}_j$. ResNet50 is chosen as the backbone encoder, which is a deep neural network that is used in the original SimSiam framework. We use a single layer MLP as the lightweight predictor. We calculate the negative cosine similarity based on Eq. (5).

Note that $\lambda$ in Eq. (4) is determined directly from Eq. (3) given the Mixup boundary and current training step. Follow notations in the previous section, denote $p_i\overset{\Delta }{=}\ h(f(x_i))$ as the predicted representation and $\tilde{z}_j\overset{\Delta }{=}\ f(\tilde{x}_j)$ as the projected embedding of $x_i$. Our training objective is to minimize the negative cosine similarity between $p_i$ and $\tilde{z}_j$ of the following form,

5 $$\begin{aligned} \mathfrak {L}_{Mixup} = -\frac{1}{2}\left( \frac{p_i}{\Vert p_i\Vert _2}\cdot \text {StopGrad}(\frac{\tilde{z}_j}{\Vert \tilde{z}_j\Vert _2}) +\frac{\tilde{p}_j}{\Vert \tilde{p}_j\Vert _2}\cdot \text {StopGrad}(\frac{z_i}{\Vert z_i\Vert _2})\right) \end{aligned},$$

where $\tilde{z}_j\overset{\Delta }{=}\ f(\tilde{x}_j)=f(\lambda (t) x_i+(1-\lambda (t))x_j)$ is the projected embedding of the mixed positive image $\tilde{x}_j$. $\lambda (t)$ is a curriculum function subject to $\lambda _{min}\le \lambda (t)\le \lambda _{max}$ for any *t* and *t* is the current training iteration. Similarly, $\tilde{p}_j\overset{\Delta }{=}\ h(\tilde{z}_j)$ represents the prediction of $\tilde{x}_j$. We perform stop gradient operation on $\frac{\tilde{z}_j}{\Vert \tilde{z}_j\Vert _2}$ and $\frac{z_i}{\Vert z_i\Vert _2}$ following the setup as in SimSiam. In summary, we replace the original augmented view $x_j$, as well as the relevant projected embedding and predicted representation, with the values corresponding to the mixed view $\tilde{x}_j$. Note that since $x_i$ is the anchor image and does not go through image mixture process, $z_i \overset{\Delta }{=}f(x_i)$ and no curriculum scheduler involved. Figure 1 illustrates the pipeline of the C-Mixup.

**Algorithm 1:**
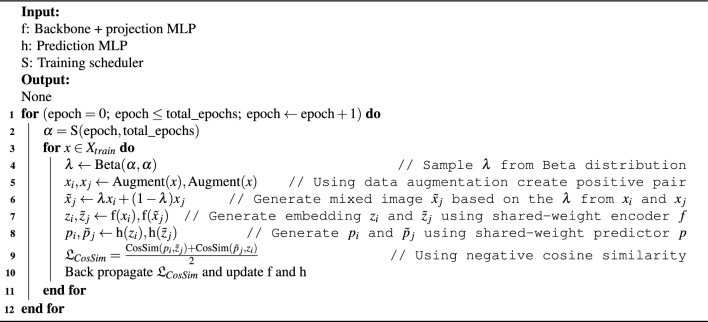
Curriculum Mixup in SimSiam.

## Dataset

**Figure 2:**
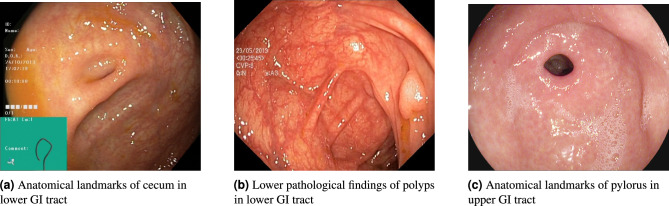
Sample images of the HyperKvasir dataset feature pathological findings and anatomical landmarks in both the upper and lower GI tract.

Our experiments are conducted using the GI endoscopic dataset, HyperKvasir^18^. In total, there are 110,079 images where 10,662 are labeled images and 99,417 are unlabeled images. The labeled dataset is collected from upper GI tract and lower GI tract consisting of 23 different classes grouped into four major categories: anatomical landmarks, quality of mucosal views, pathological findings, and therapeutic interventions. We made several modifications to the HyperKvasir dataset to accommodate our classification task. First, we trimmed ileum, hemorrhoids, ulcerative-colitis-grade-0-1, ulcerative-colitis-grade-1-2, and ulcerative-colitis-grade-2-3 in the lower GI tract and barretts and barretts-short-segment in the upper GI tract to tackle the class imbalance issue. Each class in the aforementioned 7 classes has a number of samples less than 10% of the largest class. Unlabeled dataset is used explicitly in the pre-train stage. In the fine-tuning stage, the labeled images are resized to 512 * 512 to retain as much information as possible. We randomly split the labeled image into a 4:1 ratio as training and testing data. This split setting remains the same for all experiments we conducted. Figure 2 illustrates the endoscopic images of HyperKvasir dataset.

## Experiments

In this section, we will introduce our experimental settings and results.

### Experimental settings

#### Data pre-processing

Medical images are more sensitive to color distortion than benchmark datasets such as ImageNet^63^. Therefore, instead of using the original data augmentation method as described in Simsiam^36^, we adopt the weaker color augmentation strategy from Balanced-Mixup^64^. Specifically, we first resize all inputs to 512x512 and drop strong color augmentations such as grayscale and Gaussian blur operations. Additionally, we decrease the magnitude of color jitter operations in brightness, contrast, and saturation from 0.4 to 0.25. To maintain the strength of the augmentation, we add more affine transformations. In addition to horizontal flips, we include vertical flips and randomly apply translation, rotation, or scaling to the image. It is worth noting that some of the included classes of images have a green picture in the image illustrating the position and configuration of the endoscope. We followed the experimental setting of Balanced-Mixup^64^ and included the green picture as-is in both unlabeled data for pre-training and labeled data for finetuning.

#### Simsiam with C-Mixup

In the pre-training stage, an input image will be augmented into two augmented views, view *i* and view *j*. We stochastically perform Mixup operation using Beta distribution as in Mixup^58^ on view *j* with view *i*. We use step function as our curriculum scheduler and, if not otherwise specified, we use $\alpha _{max}=\beta _{max}=0.2$ and $\alpha _{min}=\beta _{min}=1e-4$ as the Beta distribution parameter. Since $Beta(\alpha , \beta )$ exhibits a U-shape distribution when $0<\beta =\alpha <1$, we set a maximum cap of 0.5 to ensure that view *j* will always contribute the majority to the resulting mixed image, mixed view *j*. We use ResNet50 as the backbone encoder network and modify the out feature dimension of the FC layer to 2048. In addition, view *i* will go through a predictor module with with hidden dimension of 512. During the pre-training stage, the model is trained for 100 epochs on unlabeled HyperKvasir dataset, using the SGD optimizer with an initial learning rate of 0.01, weight decay set to 1e-4, and momentum set to 0.9, with cross-entropy loss. We use cosine decay learning rate scheduler for its empirical success in self-supervised tasks. The model is fine-tuned for 100 epochs with a batch size of 64 on labeled HyperKvasir dataset. The initial learning rate is set to 0.001 and weight decay is set to zero. All other hyperparameters remain the same as those used in the pre-training stage.

## Results and discussions

We utilize accuracy, F1 score, precision, and recall as performance metrics to evaluate our methods. For each experiment, we conduct 3 trials and record the corresponding results. Table 1 presents the average results for each experiment conducted. Based on the table, we can draw the following observations.

**Table 1:** Comparison of C-Mixup and baselines.

Method	Accuracy	F1	Precision	Recall	Specificity
AlexNet^65^	79.15 ± 0.3	63.13 ± 1.2	64.60 ± 1.4	64.98 ± 1.9	98.65 ± 0.2
ResNet18^14^	86.04 ± 0.2	70.67 ± 0.3	71.61 ± 0.2	71.73 ± 0.2	99.10 ± 0.1
ResNet50^14^	86.30 ± 0.3	70.75 ± 0.2	71.84 ± 0.1	72.74 ± 0.1	99.14 ± 0.1
MobileNetV2^66^	87.40 ± 0.4	71.93 ± 0.3	72.93 ± 0.5	73.35 ± 0.3	99.22 ± 0.1
VGG19^67^	82.86 ± 0.5	67.81 ± 0.7	69.22 ± 0.8	69.28 ± 0.8	98.96 ± 0.1
DenseNet121^68^	86.48 ± 0.4	68.34 ± 0.2	69.12 ± 0.3	70.02 ± 0.6	99.17 ± 0.1
ConvNeXt V2^69^	91.24 ± 0.4	83.85 ± 0.6	84.88 ± 0.9	85.00 ± 0.6	99.41 ± 0.1
EfficientNet V2^70^	91.99 ± 0.2	83.73 ± 0.3	84.60 ± 0.1	85.21 ± 0.5	99.44 ± 0.1
MaxViT^71^	92.06 ± 0.1	**84.94** ± 0.3	**85.97** ± 0.2	86.57 ± 0.3	99.41 ± 0.1
FasterViT^72^	91.65 ± 0.3	83.83 ± 0.3	84.66 ± 0.3	85.20 ± 0.1	99.46 ± 0.1
DeiT3^73^	92.31 ± 0.1	84.21 ± 0.4	85.14 ± 0.7	85.73 ± 0.5	99.53 ± 0.1
Balanced-Mixup^64^	88.04 ± 0.3	72.26 ± 0.3	73.08 ± 0.4	73.13 ± 0.3	99.27 ± 0.1
MoCov2^37^	85.74 ± 0.2	70.82 ± 0.3	71.83 ± 0.1	72.51 ± 0.2	99.08 ± 0.1
SimSiam^36^	86.87 ± 0.3	71.47 ± 0.6	72.37 ± 0.8	72.90 ± 0.9	99.19 ± 0.1
Mixup(simsiam no curriculum)	87.52 ± 0.4	71.63 ± 0.3	72.48 ± 0.3	73.27 ± 0.2	99.23 ± 0.1
C-Mixup (Batch Size=64)	88.92 ± 0.4	73.39 ± 0.3	73.68 ± 0.2	75.00 ± 0.2	99.42 ± 0.1
C-Mixup (Batch Size=256)	**92.36** ± 0.2	84.71 ± 0.5	85.32 ± 0.4	**86.63** ± 0.4	**99.57** ± 0.1

For supervised models, we fine-tuned all models using labeled endoscopic datasets with a learning rate of 0.001, and batch size of 64 and 100 epochs. We use SGD as the optimizer with momentum of 0.9 and 0 for weight decay. For self-supervised models, we first pre-train models with unlabeled endoscopic datasets with 100 epochs, then fine-tune models on labeled endoscopic datasets with the same hyperparameter setting as supervised models. For both supervised and self-supervised methods, we use ImageNet-trained weight as initialization. Our C-Mixup experiments employ an 8-step curriculum scheduler. The Mixup alpha range is from 1e-4 to 0.2. The best results are bolded.

First, among all the methods listed in Table 1, our C-Mixup consistently achieves the best performance across all evaluation metrics. In the endoscopic image classification task, C-Mixup achieves 88.92% in top-1 accuracy and 75.0% in recall. To ensure a fair comparison, we use ResNet50 as the backbone model for all self-supervised baselines. Our method achieves 2.1% over vanilla SimSiam and 2.7% over supervised ResNet50 in top-1 accuracy, and 1.9% over vanilla SimSiam and 2.6% over supervised ResNet50 in F1 score. In addition, our method outperforms Balanced-Mixup, which performs Mixup to augment virtual data samples to imbalanced classes. This means that our curriculum Mixup method is also to robust to data imbalance issue to some extent.

Second, although adding Mixup to SimSiam without curriculum design improves performance across all four evaluation metrics, it still falls short compared to Balanced-Mixup^64^. In the SimSiam + Mixup setting, we follow the original Mixup setup, where the Mixup coefficient is sampled from a Beta distribution with $\alpha =\beta =0.2$. We suspect that the challenge lies in the difficult optimization target during the initial stages of training. To test this assumption, we apply a curriculum scheduler to SimSiam, which leads to our method, C-Mixup. As a result, our observation reveals that C-Mixup significantly improves upon the Mixup method, achieving a 1.4% increase in accuracy and a 1.7% increase in F1 score. This confirms our assumption that progressively increasing the training difficulty aids in optimizing the model.

Third, our C-Mixup method surpasses all supervised learning baseline methods, including AlexNet^65^, ResNet18^14^, ResNet50^14^, MobileNetV2^66^, VGG19^67^, and DenseNet121^68^, by a significant margin. All the supervised methods are initialized with ImageNet trained weight and fine-tuned on the labeled endoscopic dataset. On the contrary, one vanilla self-supervised model, MoCoV2, which is using ResNet50 as backbone, has even lower top-1 accuracy and recall compared to supervised ResNet50. Similarly, Although vanilla SimSiam outperforms supervised ResNet50 on a small margin, it still has a lower accuracy compared to supervised MobileNetV2 in all metrics. This result indicates an undesirable pre-training outcome due to adverse noise incurred by strong data augmentation in the vanilla self-supervised setting.

### Visualization

**Figure 3:**
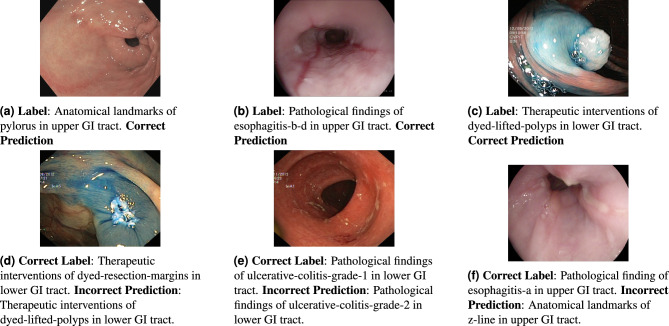
Sample images representing correct and incorrect predictions of C-Mixup. Figure (**a**–**c**) show correct predictions. Figure (**d**–**f**) show incorrect predictions.

We further visualize samples of correct and incorrect predictions to gain insights into the strengths and limitations of our method. In other cases, C-Mixup demonstrates its ability to accurately classify features in endoscopic images. In Fig. 3a, our method successfully identifies the pylorus connecting to the duodenum located in the upper GI tract. Similarly, in another example (Fig. 3b), C-Mixup detects inflammation and determines the specific type of inflammation as esophagitis-b-d, occurring in the upper GI tract. Furthermore, our method is capable of detecting therapeutic interventions performed by surgeons, such as dyed lifted polyps, as shown in Fig. 3c. These interventions are often challenging for the human eye to discern, but our method reliably identifies them.

Figure 3 illustrates examples where C-Mixup fails to classify correctly. For instance, in Fig. 3d, C-Mixup incorrectly classifies a therapeutic intervention of dyed-resection-margins in the lower GI tract as the therapeutic interventions of dyed-lifted-polyps in the lower GI tract. One possible reason for this misclassification is the bright colored bulge in the central right confuses the model with the dyed-lifted-polyp, which also tends to be reflective. In Fig. 3e, a pathological finding of ulcerative-colitis-grade-1 in the lower GI tract is incorrectly classified by C-Mixup as pathological findings of ulcerative-colitis-grade-2 in the lower GI tract. The misclassification of this example can be justified as an ambiguous stage distinction between different grades of the same medical condition. In Fig. 3f, the ground truth label indicates a pathological finding of esophagitis-a in the upper GI tract, while C-Mixup incorrectly classifies it as anatomical landmarks of the z-line, a demarcation line, in the upper GI tract. We suspect that the inflammation caused by esophagitis shown in Fig. 3f is of early stage and not conspicuous.

**Figure 4:**
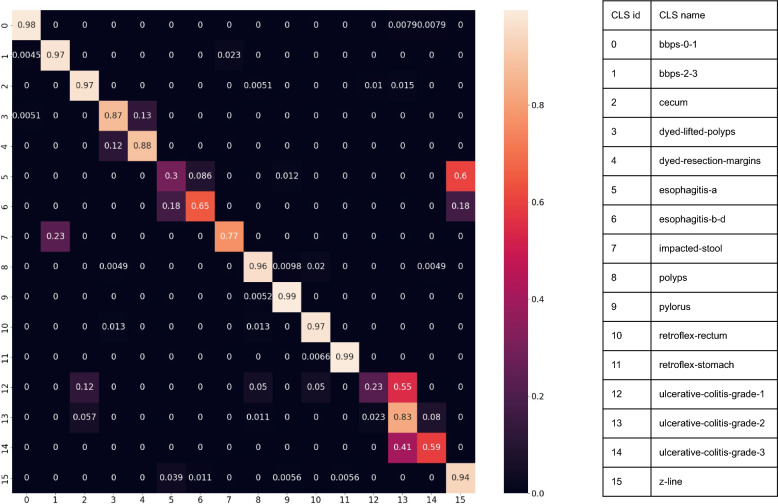
Confusion matrix of C-Mixup 8 step on all 16 classes.

Figure 4 shows the confusion matrix of C-Mixup of all 16 classes. It is notable that class ulcerative-colitis-grade-1, ulcerative-colitis-grade-2, and ulcerative-colitis-grade-3 are most confusing to the model by which a significant portion of grade 1 and 3 ulcerative colitis are misclassified as grade 2. By visual inspection, we found that the data augmentation in the pretraining stage might create some distortion to the image, therefore impacting the model’s performance in rating the severity of ulcerative colitis. In the real world, assessing the severity of ulcerative colitis is a complicated procedure involving multiple tests^74^. While the C-Mixup model is not able to achieve superior performance in distinguishing the severity of ulcerative colitis, it does classify ulcerative colitis with a low error rate on other classes. Another observation is that C-Mixup has inferior performance in distinguishing between z-line, a faint zig-zag impression that demarcates the transition site between the esophagus and the stomach, and esophagitis-a, an inflammation of the esophagus. Esophagitis-a is the least severe esophagitis included in the dataset, and since it appears in the esophagus which z-line might present, it is possible that both esophagitis and z-line appear. Our visual inspection confirms this observation and therefore the misclassification is explainable.

### Ablation study

In this section, we perform ablation studies on various aspect of C-Mixup to better understand each component of our proposed method.

#### Curriculum schedulers

In our proposed C-Mixup method, we employ a simple step function as the curriculum scheduler. However, in this section, we aim to investigate how different scheduler functions impact the performance of C-Mixup. Specifically, we want to determine if our method is sensitive to the choice of curriculum scheduler. To explore this, we examine both discrete and continuous curriculum schedulers.

**Figure 5:**
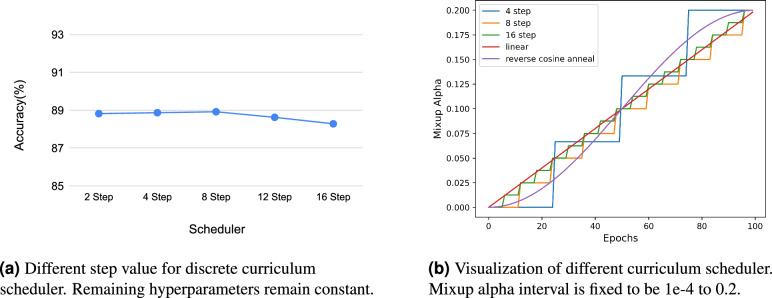
Ablation on scheduler.

For discrete curriculum schedulers, we experiment with step functions using step sizes of 2, 4, 8, 12, and 16. Figure 5a illustrates the top-1 accuracy for different step values. Our findings reveal that C-Mixup is robust to changes in the step value, confirming that a gradual increase in Mixup lambda results in improved performance compared to using Mixup without a curriculum setup.

In the case of continuous curriculum schedulers, we investigate linear and reverse cosine annealing functions. Figure 5a provides a visualization of how Mixup alpha changes with respect to different curriculum schedulers, while Table 2 presents the performance metrics of various curriculum schedulers. Results indicate that the cosine anneal scheduler achieves the best performance, with a top-1 accuracy of 89.31%. We attribute this success to the fact that cosine annealing mirrors the way humans learn in real life. We start with easier tasks and gradually increase the difficulty as learning progresses. Throughout this process, the majority of the training time is dedicated to the initial and final stages, allowing the model to establish a strong foundation and effectively tackle challenging tasks. This strategy proves to be highly effective, as evidenced by the superior performance of the reverse cosine anneal scheduler across all evaluation metrics.

**Table 2:** Experiments with different curriculum schedulers.

Epoch	Curriculum scheduler	Accuracy	F1	Precision	Recall	Specificity
100	2 step	88.82 ± 0.2	72.30 ± 0.3	72.53 ± 0.2	74.24 ± 0.3	99.28 ± 0.1
	4 step	88.87 ± 0.3	71.91 ± 0.4	72.23 ± 0.4	73.87 ± 0.2	99.30 ± 0.1
	8 step	88.92 ± 0.4	73.39 ± 0.3	73.68 ± 0.2	75.00 ± 0.2	**99.42 ± 0.1**
	12 step	88.62 ± 0.3	72.26 ± 0.3	73.04 ± 0.6	74.23 ± 0.3	99.27 ± 0.1
	16 step	88.28 ± 0.4	71.95 ± 0.5	72.53 ± 0.3	73.64 ± 0.6	99.23 ± 0.1
	linear	89.06 ± 0.6	**73.68 ± 0.6**	73.92 ± 0.7	**75.13 ± 0.4**	99.32 ± 0.2
	cosine anneal	**89.31 ± 0.7**	73.56 ± 0.9	**74.14 ± 0.8**	74.97 ± 0.6	99.39 ± 0.2

Experiments are conducted in Mixup alpha interval 1e-4 to 0.2. Best results are bolded.

#### Mixup interval

Since C-Mixup dynamically and progressively determines the Mixup alpha, another critical component of our proposed method is the Mixup alpha interval. In Table 1 and Fig. 5b, we fixed the Mixup interval to range from 0 to 0.2. However, it is important to investigate the optimal Mixup intervals. As the actual Mixup $\lambda$ is sampled from a Beta distribution parameterized by the Mixup alpha, we set the minimum boundary of the Mixup alpha interval as 1e-4. Additionally, since a Beta distribution with parameters $\alpha =\beta < 1$ exhibits a U-shape, we capped the maximum boundary of the Mixup alpha interval to be smaller than 0.5. We examined three different intervals: 1e-4 to 0.2, 0.2 to 0.4, and 1e-4 to 0.4. The complete results are presented in Table 3.

**Table 3:** Performance of different Mixup alpha interval.

Min Mixup alpha	Max Mixup alpha	Accuracy	F1	Precision	Recall	Specificity
1e-4	0.2	**88.92 ± 0.4**	**73.39 ± 0.3**	**73.68 ± 0.2**	**75.00 ± 0.2**	**99.42 ± 0.1**
0.2	0.4	88.87 ± 0.3	72.85 ± 0.3	73.13 ± 0.2	74.43 ± 0.1	99.35 ± 0.1
1e-4	0.4	88.67 ± 0.5	71.69 ± 0.4	71.88 ± 0.2	73.86 ± 0.2	99.20 ± 0.1

Experiments performed in this table are using C-Mixup 8-step. All hyperparameters other than Mixup interval are fixed. Best results are bolded.

From the results, several observations can be made. Firstly, increasing the maximum Mixup alpha to 0.4 led to a 1.7% decrease in F1 score, accompanied by lower precision and recall. This decrease in performance can be attributed to a wider distribution of $\lambda$ resulting from a larger Mixup alpha interval, which introduces turbulence in the training process. Specifically, it caused a 1.6% decrease in precision and a 1.2% decrease in recall. Secondly, the Mixup interval with a larger Mixup alpha (0.2 to 0.4) exhibited subpar performance. This can be explained by the fact that a higher Mixup alpha increases the likelihood of the mixed image $\tilde{x}_j$ containing a larger portion of $x_i$, leading to more trivial image pairs of $x_i$ and $\tilde{x}_j$.

## Conclusion

In this paper, we propose C-Mixup, a self-supervised learning framework that leverages curriculum Mixup on SimSiam to utilize a large unlabeled endoscopic dataset. Our method aims to mitigate the negative impact of additive noise caused by strong data augmentation by incorporating curriculum learning. We innovatively combine the concepts of curriculum learning and Mixup to create a progressive data augmentation framework that enhances the pre-training of SimSiam on endoscopic datasets. Our empirical results demonstrate that C-Mixup outperforms both supervised and self-supervised baselines, achieving an impressive top-1 accuracy of 88.92% and an F1 score of 73.39%. We also conducted several ablation settings to further explore the potential of our proposed method. The results strongly suggest that our curriculum Mixup can serve as a reliable aid in detecting gastrointestinal diseases using endoscopy.

## Data Availability

All experiments are carried out using the publicly available HyperKvasir^18^ dataset with simple modification described in Dataset section.
